# A unique strategy for large-bowel perforation with ventriculoperitoneal shunt: Conversion to ventriculoatrial shunt: A case report

**DOI:** 10.1016/j.ijscr.2019.10.034

**Published:** 2019-10-22

**Authors:** Shota Akabane, Hirokazu Iijima, Yukari Kobayashi, Kazunao Watanabe

**Affiliations:** Department of General Surgery, Tokyo-Nishi Tokushukai Hospital, 196-0003, Matsubara 3-1-1, Akishima, Tokyo, Japan

**Keywords:** CSF, cerebrospinal fluid, CNS, central nervous system, CTRX, ceftriaxone sodium hydrate, MEPM, meropenem hydrate, VCM, vancomycin hydrochloride, Case report, Bowel perforation, Central nervous system, Meningitis, Peritonitis, Ventriculoperitoneal shunt

## Abstract

**Introduction:**

Large-bowel perforation can lead to critical sepsis, and urgent intervention including surgery is indispensable to control systemic infection. Here, we describe a strategy for large-bowel perforation using a ventriculoperitoneal shunt.

**Case presentation:**

A 74-year-old Japanese female with a history of cerebral aneurysm clipping and ventriculoperitoneal shunting due to aneurysmal subarachnoid hemorrhage presented with lower abdominal pain, fever, and disturbed consciousness. Clinical findings indicated a diagnosis of large-bowel perforation and ventriculoperitoneal shunt-transmitted bacterial meningitis. Thus, sigmoidectomy and shunt externalization were performed, and the ventriculoperitoneal shunt was converted to a ventriculoatrial one.

**Conclusion:**

Based on our experience and the literature, we successfully discuss bowel perforation management with respect to the ventriculoperitoneal shunt, including the utility of the ventriculoatrial shunt as an alternative.

## Highlights

• Large-bowel perforation can lead to critical sepsis, and urgent intervention including surgery is indispensable to control systemic infection.
• We describe a strategy for large-bowel perforation using a ventriculoperitoneal shunt that was converted to a ventriculoatrial shunt and discuss its utility based on the literature.

## 1 Introduction

Because large-bowel perforation can cause critical sepsis, urgent intervention, including surgery, is indispensable for controlling systemic infection. Various conditions, such as diverticular diseases, neoplasms, and inflammatory diseases, can lead to spontaneous large-bowel perforation. Ventriculoperitoneal (VP) shunts have been used to relieve brain pressure caused by cerebrospinal fluid (CSF) accumulation and have been applied to patients with normal pressure hydrocephalus or bypass obstruction of CSF flow due to incidents such as subarachnoid hemorrhage. A systematic review reported a pooled mean internalized shunt infection rate of 5%–15% [1]; although these infections are usually diagnosed during the initial month after replacement, regardless of placement duration, they are rarely caused by peritonitis or bowel perforation.

Here, we describe a strategy for large-bowel perforation using a VP shunt that was converted to a ventriculoatrial (VA) shunt and discuss its utility based on the literature.

This case report has been reported in line with the SCARE criteria [2].

## 2 Case presentation

A 74-year-old Japanese female presented with lower abdominal pain, fever, and mental status changes that extended for 4 or 5 days. She complained of intermittent vomiting that began just before she arrived at the hospital. Her medical history included surgical clipping due to aneurysmal subarachnoid hemorrhage (SAH) and VP shunting that was performed 3 years ago. Physical examination revealed mental disturbance (Glasgow Coma Scale: E3V4M6) and a body temperature of 39.5 °C. She also exhibited lower abdominal tenderness with distention and muscular defense. No signs of meningeal irritation, such as nuchal rigidity, were observed.

Laboratory examination revealed drastically elevated levels of inflammatory markers and anemia [white blood cell (WBC), 22,000/μl; Hbg, 10.6 g/dl; and C-reactive protein (CRP), 17.93 mg/dl], and CSF obtained via lumbar puncture demonstrated no notable abnormalities (WBC, 2/μl; protein, 40 mg/dl; and glucose, 50 mg/dl).

**Fig. 1:**
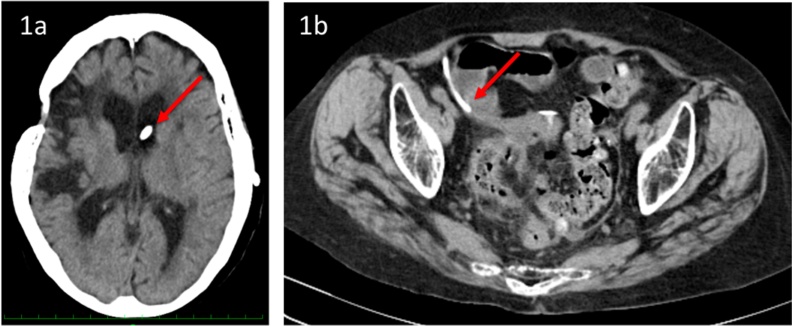
a) Head computed tomography (CT) revealed a low-density area in the right hemisphere indicating impairment due to aneurysmal subarachnoid hemorrhage, and the ventricular part of the ventriculoperitoneal (VP) shunt was inserted into the left ventricle (Red arrow). B) Abdominal CT revealed a diverticular sigmoid colon surrounded by free air and isodensity ascites, and the peritoneal part of the VP shunt was placed far from the sigmoid colon (Red arrow).

Head computed tomography (CT) revealed a low-density area in the right hemisphere of the brain, indicating impairment due to SAH. The proximal end of the VP shunt was inserted into the left ventricular part (Fig. 1a). Abdominal CT revealed a diverticular sigmoid colon surrounded by free air and isodense ascites (Fig. 1b). However, the distal end of the VP shunt was placed far from the sigmoid colon. The patient was diagnosed with generalized peritonitis due to perforated diverticulum and was suspected to have VP shunt-transmitted meningitis. Exploratory laparotomy, including sigmoidectomy and colostomy installation, was performed after laparoscopic examination. The peritoneal side of the VP shunt was resected at the subclavian level and temporarily externalized to determine central nervous system (CNS) infections and CSF output amount. A perforated diverticulum, which was far from the peritoneal side of the VP shunt, was intraoperatively detected in the sigmoid colon. Pathological findings revealed no evidence of malignancy.

For two days postoperatively, the patient was administered broad-spectrum antibiotics (meropenem hydrate, 4 g/day; vancomycin hydrochloride, 1.5 g/day) in addition to endotoxin absorption therapy using Toraymixin® (TORAY MEDICAL, Japan) as part of her postoperative course. Based on the results of two sets of blood cultures that detected *Escherichia coli*, antibiotics were de-escalated and ceftriaxone sodium hydrate (4 g/day) was initiated.

In terms of CNS infection, CSF was analyzed on the third, fifth, and tenth days postoperatively; the results were negative for culture and within normal range for glucose or cell counts. Her mental status had fairly improved postoperatively, implying that her mental disturbance was caused by sepsis rather than CNS infection.

**Fig. 2:**
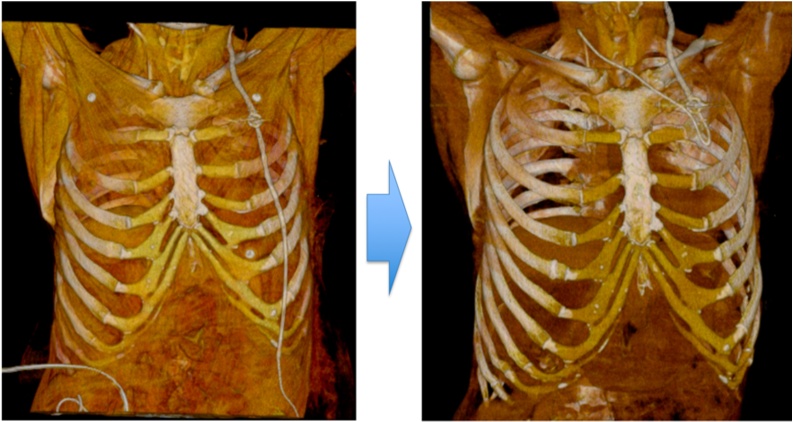
The catheter inserted into the right atrium via the right jugular vein under C-arm fluoroscopy was connected to the reservoir of the original ventriculoperitoneal (VP) shunt via a subcutaneous tunnel.

On postoperative day 14, the mean output of drained CSF was approximately 300 ml/day, indicating that the patient was still shunt-dependent. CSF analysis revealed no evidence of infection; therefore, the externalized ventricular shunt was converted to a VA shunt. The catheter contained in the X-PORT™ isp implantable Port (C. R. Bard, USA) was used as an alternative for the VA shunt at the atrial side. The catheter inserted into the right atrium via the right jugular vein under C-arm fluoroscopy was connected to the reservoir of the original ventricular side of VP shunt via a subcutaneous tunnel (Fig. 2).

The patient’s postoperative course was uneventful, and she was discharged on the 30^th^ day postoperatively without major complications.

## 3 Discussion

Our case highlights two important considerations. First, large-bowel perforation with a VP shunt can be critical and requires urgent intervention. Second, to our best knowledge, this is the first report describing VP shunt converted to a VA shunt for preventing CNS infection transmitted by the former.

Shunt-related infection commonly occurs as complication with an incidence rate of 5.6%–12.9%, and such infections are most likely diagnosed during the initial month after shunting [3]. In contrast, spontaneous bowel perforation complicated by a VP shunt is rare, but potentially fatal, and can occur any time after shunting in approximately 0.01%–0.07% of patients [4,5]. This infection is likely caused by VP shunts’ sharp ends, local inflammatory reactions or fibrosis around the distal catheter resulting in pressure on the bowel area, shunt-related chronic irritation, previous adhesions or infections, or silicone allergy due to foreign body [4,6]. Mortality rate after perforation was relatively high (15%–18%) and further increased when infection occurred in 22% of patients with CNS infection and 33% with intra-abdominal infection [[7], [8], [9]]. Bowel-perforation treatment complicated with VP shunts depends on the clinical presentation. Peritonitis, intraperitoneal abscess, and sepsis should always be treated with exploratory laparotomy, lavage, primary bowel-wall closure, and shunt removal [4,8]. The ventricular part should be drained for at least 14 days, with antibiotic treatment as prophylaxis [4,10]. Once CSF cultures are repeatedly negative, a new peritoneal shunt catheter can be placed at the opposite site [10].

VA shunts have been commonly used as the second-line treatment for hydrocephalus when the peritoneum is unsuitable for distal catheter placement [11]. Yet, VA shunting may be underutilized due to neurosurgeons’ technical preferences, biases, and concerns regarding cardiopulmonary complications; most of those complications have been reported in adult patients who underwent VA shunting during childhood [12].

In our case, large-bowel perforation was seemingly caused by diverticulum perforation and not irritation due to the shunts’ peritoneal end. The patient was determined to be shunt-dependent based on her daily CSF output; therefore, the externalized ventricular shunt was secondarily converted to a VA shunt. Although total shunt replacement more reliably eliminates infection sources, shunt resection at the subclavian level and its ventricular end still remains controversial. However, multiple ventricular punctures may confer risk of choroidal hemorrhage and intra-abdominal adhesion after bowel perforation and can interfere with safe shunt revision [10]. Although definitive criteria to evaluate the risk of shunt-transmitted CNS infection are lacking, CSF analysis, including culturing, was performed on the 3rd, 5th, and 10th days postoperatively; results revealed no evidence of infection; thus, the possibility of CNS infection was excluded.

## 4 Conclusion

Because large-bowel perforation with a VP shunt can be critical and urgent interventions are indispensable, VP shunts can be converted to VA shunts to prevent shunt-transmitted CNS infection.

## Sources of funding

The authors declare that they have no sources of funding.

## Ethical approval

All patients received an explanation of the procedures and possible risks of the study, and gave written informed consent. This study was reviewed and ethical approval has been exempted by Tokyo West Tokushukai Hospital Institutional Review Board.

## Consent

Written informed consent was obtained from the patient’s next of kin for publication of this case report and accompanying images. A copy of the written consent is available for review by the Editor-in-Chief of this journal on request”.

## Author’s contribution

Shota Akabane: study concept, writing the paper.

Hirokazu Iijima: study concept, review and revision of the paper.

Yukari Kobayashi: review and revision of the paper.

Kazunao Watanabe: review and revision of the paper.

## Registration of research studies

A unique strategy for large-bowel perforation with ventriculoperitoneal shunt: Conversion to ventriculoatrial shunt.

researchregistry1087.

## Guarantor

Kazunao Watanabe.

## Provenance and peer review

Not commissioned, externally peer-reviewed.

## Declaration of Competing Interest

The authors declare that they have no conflicts of interest.
